# Functional Recovery after Intramyocardial Injection of Adipose-Derived Stromal Cells Assessed by Cardiac Magnetic Resonance Imaging

**DOI:** 10.1155/2021/5556800

**Published:** 2021-04-22

**Authors:** Janusz Konstanty-Kalandyk, Jerzy Sadowski, Anna Kędziora, Małgorzata Urbańczyk-Zawadzka, Jakub Baran, Paweł Banyś, Bogusław Kapelak, Jacek Piątek

**Affiliations:** ^1^Department of Cardiovascular Surgery and Transplantology, John Paul II Hospital, Krakow, Poland; ^2^Jagiellonian University Medical College, Krakow, Poland; ^3^Department of Radiology and Diagnostic Imaging, John Paul II Hospital, Poland; ^4^Department of Interventional Cardiology, John Paul II Hospital, Krakow, Poland

## Abstract

**Aims:**

A major clinical concern is the continuous increase in the number of patients diagnosed with advanced coronary artery disease, ischemic heart failure, and refractory angina, and one of the most promising treatment options for these conditions is stem cell-based therapy. The aim of this study was to assess the functional improvement following intramyocardial injection of adipose-derived stromal cells, using cardiac magnetic resonance.

**Methods and Results:**

Thirteen patients with ischemic heart failure, reduced left ventricular ejection fraction, refractory angina, and who have been disqualified from any form of direct revascularization were enrolled in the study with transthoracic autologous adipose-derived stromal cell implantation. All patients underwent cardiac magnetic resonance prior to the procedure and after 12 months of follow-up. A significant increase in stroke volume (83.1 ± 8.5 mL vs 93.8 ± 13.8 mL, *p* = 0.025) and stroke volume index (43.3 ± 7.6 mL/m^2^ vs 48.7 ± 9.1 mL/m^2^, *p* = 0.019), a statistical trend toward an increase in left ventricle ejection fraction (36.7 ± 13.2 vs 39.7 ± 14.9, *p* = 0.052), and cardiac output improvement (5.0 ± 0.7 vs 5.5 ± 0.9, *p* = 0.073) was observed in the patient postprocedure. Enhanced relative regional thickening was noted in the segments with adipose-derived stromal cell implantation.

**Conclusions:**

Intramyocardial adipose-derived stromal cell implantation is a promising therapeutic option for selected, symptomatic patients with ischemic heart failure, who have preserved myocardial viability despite being unsuitable for direct revascularization.

## 1. Introduction

Ischemic heart disease, caused by the reduction of blood supply in the myocardium, has been a major clinical burden of the recent years. Cardiovascular diseases have contributed to the highest number of years of life lost in patients across the world [1, 2]. Obstructions of the large- and medium-sized coronary arteries are frequently amenable to surgical (coronary artery bypass grafting: CABG) or endovascular (percutaneous coronary intervention: PCI) revascularization procedures with short- and long-term benefits. Nevertheless, advanced coronary artery disease (CAD) can lead to heavy calcifications in the arteries and make the patient ineligible for direct revascularization [3–5].

Ischemic heart failure (IHF) is characterized by typical symptoms of breathlessness, ankle swelling, and fatigue, with CAD as the underlying cause [6]. Currently, a large number of patients, diagnosed with IHF or refractory angina, are disqualified from direct revascularization, and the estimated morbidity, among septa- and octogenarians, is approximately 10% [7–9]. These “no option” patients have a significantly impaired quality of life and limited treatment options [5, 10]. Moreover, a clear relationship between the severity of symptoms and survival has been proven in nearly 20% of cases, using 12-month all-cause mortality rates [11]. However, noninvasive imaging of myocardial viability should be performed to assess eligibility for revascularization procedures, and cardiac magnetic resonance (CMR) is extremely efficacious indistinguishing irreversible injury from myocardial hibernation [12].

Based on current treatment guidelines, novel therapies, including promotion of collateral growth, transmural redistribution of blood flow, and neuromodulation of the cardiac pain syndrome, are considered for these individuals, and among these, stem cell-based therapies are the most promising treatment strategy [13]. The formation of new blood vessels, a target for stem cell-based therapies, is a complex and integrated process for neovascularization that involves angiogenesis, vasculogenesis, and arteriogenesis. In angiogenesis, new microvessels are generated by the proliferation and migration of endothelial cells from preexisting vasculature [14]. Adipose-derived stromal cells (ADSCs) have been previously assessed in a rat model by Wang et al. [15], with promising results of cardiac function improvement, ventricular remodeling prevention, and angiogenesis enhancement in the infarct border zone.

Nevertheless, evidence regarding the clinical effectiveness of stem cell-based therapies is still limited [16]. In the present study, we analyzed results from a 12-month follow-up of cardiac function, assessed using cardiac resonance imaging, following autologous ADSC intramyocardial implantation.

## 2. Methods

### 2.1. Study Population

Fifteen patients with IHF were enrolled in this study in accordance with a previously published study protocol [17, 18].

Briefly, the inclusion criteria were symptomatic IHF with or without reduced left ventricular ejection fraction (LVEF) (NYHA class II or III), stable angina (CCS class II or greater), and ineligibility for direct revascularization (PCI or CABG) as assessed by the local Heart Team. Exclusion criteria included LVEF less than 20%, acute myocardial infarction within 6 months before enrollment, history of malignancy, renal dysfunction (glomerular filtration rate<30 mL/min/1.73m^2^), unexplained hematological abnormalities, existing valvular heart disease, requirement of surgery, congenital heart disease, severe arrhythmias, contraindications for general anesthesia, and inability to perform CMR imaging (owing to claustrophobia or allergy to contrast).

All patients signed the informed consent form. Thirteen patients completed the 12 months follow-up, including the CMR follow-up assessment. Two patients resigned from the study before the final follow-up appointment.

The average age in the study group was 65 years, and the mean LVEF was 36.7% (±13.2) prior to the procedure. All patients had previous myocardial infarction; before enrollment, 5 of the 13 patients (38%) had undergone PCI, and 3 of the 13 patients (23%) had undergone CABG (Table 1).

### 2.2. Cell Preparation and Implantation

Patients were placed under general anesthesia, and fat tissue (liposuction) was collected from the abdominal area; automatic suction was not used to collect body fat. Subsequently, the adipose tissue was subjected to purification using the Celution® 800/CRS System (Cytori Therapeutics, San Diego, CA), according to the manufacturer's instructions, to obtain ADSCs, and 5 mL of concentrated stem cell suspension was obtained from the patients. For cell purification, we used dedicated medical equipment that enables reliable and reproducible isolation of ADSCs from the fat tissue [19]. The anterior heart wall was visualized via left-sided minithoracotomy, and the average number of implanted stem cells was 40 × 10^6^.The ADSC implantation site was determined by the position of the mid-left anterior descending (mid-LAD) artery. The harvested stem cell suspension was then immediately delivered with a PHOENIX combined delivery system (CryoLife, USA), and 1 mL of suspension was applied around each single channel created using the laser. No perioperative complications were reported, and the patients were discharged home on postoperative day 7. The detailed protocol for ADSC implantation has been described previously [17, 18].

### 2.3. CMR: Imaging Protocol

Breath-hold, ECG-gated CMR was performed using the 1.5 T scanner Magnetom Sonata Maestro Class (Siemens, Erlangen, Germany) equipped with a cardiac phased array coil. Cine and morphologic imaging was performed in the left ventricular two-chamber, four-chamber, apical long-axis, and short-axis view, encompassing the entire left ventricle (LV). Cine imaging was performed using a balanced steady-state free precession gradient echo technique (slice thickness 8 mm, no gap, matrix 256 × 192, in-plane resolution 1.3 × 1.3 mm, TR/TE 39/1.1 ms, flip angle 59°). Ten minutes after intravenous infusion of 0.15 mmol/kg body weight gadobutrol (Gadovist, Bayer Schering Pharma, Berlin, Germany), late gadolinium-enhanced (LGE) imaging was performed using T1-weighted segmented inversion-recovery pulse sequence (slice thickness 8 mm, no gap, matrix 256 × 192, in-plane resolution 1.3 × 1.3 mm, TR/TE 650/4.9 ms, flip angle 30°, TI set to null normal myocardium).

### 2.4. CMR: Image Analysis

Cine and LGE images were assessed offline using dedicated software (MASS Medis, Leiden, Netherlands) by an independent observer, blinded to the clinical data.

Cine images: endocardial and epicardial LV borders were outlined using the short-axis images to calculate end-diastolic volume (EDV), end-systolic volume (ESV), stroke volume (SV), myocardial mass, and EF.

LGE images: qualitative assessment for the presence and location of hyperintense lesions in contrast to hypointense viable myocardium was performed using the LGE images. The number of affected segments was calculated, and the transmurality of myocardial wall thickness was scored using the LGE images as follows: 0 (no LGE), 1(1–25%), 2 (26–50%), 3 (51–75%), or 4 (76–100%).

### 2.5. Health Status and Quality of Life

Health status was evaluated on the basis of the Short Form Health Survey (SF-36), which is commonly used in cardiac rehabilitation studies [20]. Scores for physical functioning (PF), role-physical (RP), bodily pain (BP), general health (GH), vitality (VT), social functioning (SF), role-emotional (RE), and mental health (MH) were calculated separately at baseline, 6 months and 12 months after the procedure with 100 as a maximum for quality of life in each category.

### 2.6. Statistical Analysis

Quantitative findings are presented as the mean ± standard deviation or median (interquartile range) when appropriate. Qualitative findings are presented as raw numbers and/or percentages. Statistical tests performed for related samples were paired *t*-test or Wilcoxon signed-rank test, when appropriate. The Friedman test was used to analyze changes in quality of life over the observation period. For categorical variables, McNemar's test was applied. Statistical analysis was performed using STATISTICA 13.3 (StatSoft, Tulsa, OK, USA). A *p* value <0.05 was considered statistically significant.

This study was funded by the Polish National Science Centre (contract number UMO-2011/03/B/NZ5/01437). This article was supported by the science fund of the John Paul II Hospital, Cracow, Poland (no. FN5/2021 to J.K.K.), and Jagiellonian University Medical College. The study was approved by the Bioethics Committee at the regional medical chamber in Krakow, Poland (approval No. 115/KBL/OIL/2012).

## 3. Results

**Figure 1:**
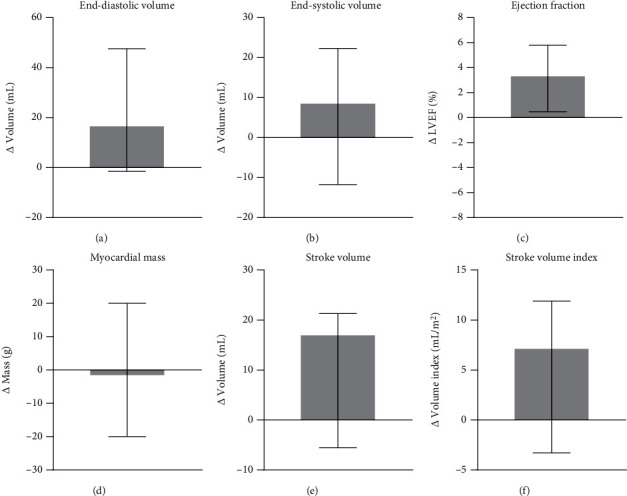
Changes in the left ventricle function assessed by cardiac magnetic resonance. (a) LVEDV: left ventricle end-diastolic volume, (b) LVESV: left ventricle end-systolic volume, (c) LVEF: left ventricle ejection fraction, (d) myocardial mass, (e) stroke volume, and (f) stroke volume index.

Preoperatively, all subjects had impaired the left ventricular systolic function. Twelve months postprocedure, a significant improvement was noted in SV (83.05 mL preprocedure vs 93.83 mL postprocedure; *p* = 0.025) and stroke volume index (EDV; 43.27 mL/m^2^ preprocedure vs 48.72 mL/m^2^ postprocedure; *p* = 0.019; Figure 1, Table 1). Additionally, LVEF slightly increased when compared to the baseline level (36.72% preprocedure vs 39.69% postprocedure; *p* = 0.052; Figure 1; Table 2). Importantly, the higher SV/SVi resulted from generally unchanged EDV, or EDV index (EDVi), and reduced ESV of the LV (Table 2). LV recovery was also observed in terms of increased cardiac output and cardiac index (CI; 2.6 L/min/m^2^ vs 2.9 L/min/m^2^; *p* = 0.075; Table 2). Myocardial mass remained stable throughout the study.

**Figure 2:**
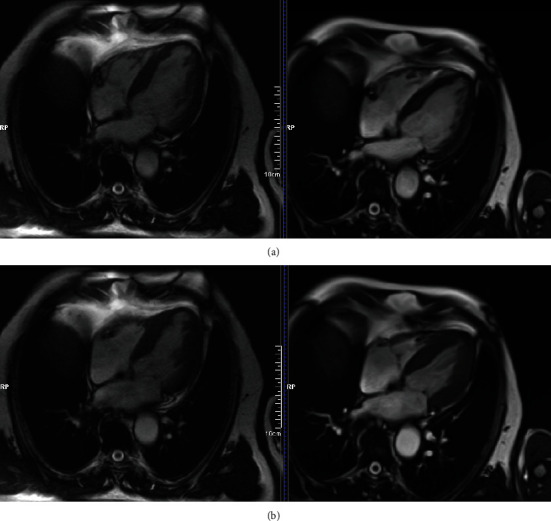
Cardiac magnetic resonance assessment at baseline and at 12 months follow-up. 71 years old male, cine SSFP (steady-state free precession) 4 chamber view—diastolic (a) and systolic (b) phase. LVEF 43% vs 54% at 12 months follow-up.

Additionally, analysis of the CMR volume–time curve did not show any change in peak ejection rate index (PERi), which is the ratio of PER to EDV of the LV, peak filling rate index (PFRi), which is the ratio of PFR to EDV of the LV, and peak ejection time (Table 2). Myocardial thickening improved in the segments where ADSCs were delivered and in regions supplied by the LAD artery (Table 3). CMR findings included improved inwards movement and myocardial thickening in ventricular septum, corresponding with LVEF increase (Figure 2).

**Figure 3:**
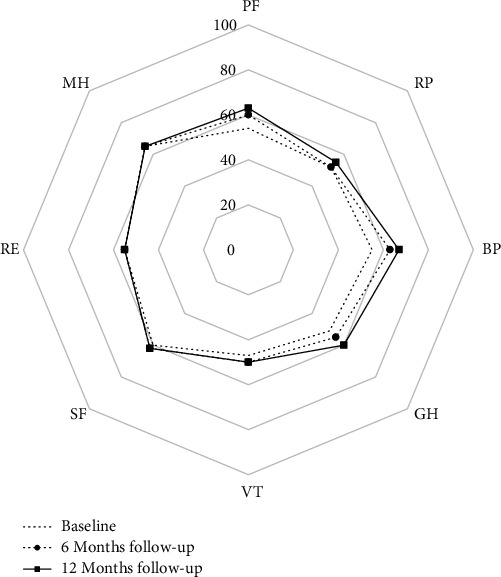
Short Form Health Survey (SF-36) results at baseline, 6 months, and 12 months after ADSC implantation. PF: physical functioning; RP: role-physical; BP: bodily pain; GH: general health; VT: vitality; SF: social functioning; RP: role-emotional; MH: mental health.

Within the 12 months follow-up, no deaths and no adverse events related to ADSCs implantation were reported. Two patients (15.4%) required hospital readmissions due to cardiac causes (HF symptoms exacerbation with successful medical therapy optimization). The quality of life improved over the 12 months after the procedure in terms of PF (*p* < 0.001), BP (*p* < 0.001), and GH (*p* < 0.001) (Figure 3).

## 4. Discussion

CMR is extremely efficacious in evaluating myocardial ischemia and myocardial function in patients with heart failure (HF) and CAD [6]. Although previous studies have shown the effectiveness of stem cell-based therapies in terms of overall functional recovery [21, 22], to our knowledge, this is the first to show the improvement in myocardial viability of treated regions by using a precise protocol of CMR-based assessment. Enhanced myocardial thickening was observed at the ADSC implantation site, in contrast to that in untreated regions, where a decrease in relative thickening was noted (Table 3; Figure 2). A vast improvement was observed in regions directly treated (segments 7, 11, 12, 13, and 16) or supplied by the LAD artery (segments 1, 2, 7, 8, 13, 14, and 16), which was the blood vessel nearest to the implantation site. Recovery was also noted in regions (segments 6, 12, 15, and 16) located at a distance from the implantation site and are supplied by the LAD artery (Table 3). Therefore, the vast recovery, not limited to the anterior wall segments, underlines the therapeutic benefit of ADSC implantation.

Despite the small sample size, a significant improvement in the LV systolic function was noted (Figure 1). Additionally, EDV and ESV of the LV are of prognostic value in HF management [23]; therefore, the observed stability in EDV and slight reduction in ESV of the LV over the 12 months of follow-up suggests a potential inhibition of LV remodeling progression. Similar assessment of efficacy endpoints is being used in the Danish Multicenter study with a 6-month follow-up period and a double-blind, placebo-controlled protocol [24]. However, the present study provided novel adjunctive indicators of the systolic and diastolic function, such as PERi and PFRi, with minor changes observed in them. These indicators, consisting of the classic and our novel indicators, address LV functional improvement following treatment.

The observed enhanced myocardial thickening and left ventricle function correspond with initial 6 months results reported for this trial [17]. Previously, we described alleviation in terms of angina and increase in LVEF assessed by transthoracic echocardiography. Similarly, significant improvement in health status and quality of life, based on patients' self-evaluation with SF-36 questionnaire, was noted over the 1 year follow-up period.

Moreover, hospital readmissions in patients with HF remain a great health care problem, reaching the annual rate of 41% based on the recently published data for the Polish population [25]. In our study group, only 2 patients (15.4%) required a hospital readmission due to cardiac causes within 12 months after the procedure. Medical therapy optimization allowed to alleviate the symptoms, and patients were discharged home after 7 and 10 days, respectively.

Undoubtedly, stem cell-based therapies are still evolving and require further investigation. However, as no adverse events related to ADSC implantation were reported within 12 months follow-up in this study, the proposed procedure can be considered a safe treatment option for highly symptomatic patients. ADSCs have been previously investigated in the Athena Trial; however, the percutaneous delivery protocol was associated with neurological complications, resulting in premature termination [26]. The transthoracic approach, proposed in our study, allowed for a safe and precise delivery in the desired area [18]. Nevertheless, the results of the largest, international, multicenter, double-blind, and placebo-controlled study assessing the efficacy of direct intramyocardial ADSC injection (SCIENCE) are still awaited [27].

Several stem cell lines have been previously investigated, and the results from the mesenchymal stromal cell therapy in heart failure (MSC-HF) trial have been recently published [28]. The MSC-HF trial was a randomized, double-blind, placebo-controlled, trial investigating intramyocardial injections of autologous bone marrow-derived mesenchymal stromal cells (MSCs) in patients with IHF. The assessed endpoints and CMR protocol were similar to those in our study, and the observed LV functional improvement following MSC injections is similar to our findings with a different stem cell line. ADSCs were chosen for this study because of their ability to differentiate into different cell lineages and for their therapeutic effects relying on paracrine secretion. The stromal fraction of adipose tissue contains a multicellular population of a high proportion of MSCs and CD34+ stem cells. Therefore, ADSCs show a stronger proliferative potential than bone marrow-derived stem cells [14, 29, 30], making ADSC implantation a prominent treatment option.

The major limitations of our study include small sample size and lack of a control group. It should be noted that all subjects received optimal medical therapy at least 12 months before the operation and continued treatment throughout the follow-up period. Therefore, the study design does not allow for definite conclusions regarding the effectiveness of the applied treatment. Nevertheless, the study provides novel information, including successful implementation of the CMR myocardial viability assessment protocol.

In conclusion, an appropriate qualification protocol with CMR-assessed viability can aid the selection of the best candidates for indirect revascularization treatments, in terms of safety and efficacy. Symptomatic patients with IHF, ineligible for direct revascularization, selected using a CMR-based assessment, can consider transthoracic ADSC injection as a safe alternate treatment option for angina symptoms and improvement in LV viability.

## Figures and Tables

**Table 1:** Baseline assessment.

Baseline characteristics	*n* = 13
IHF, *n* (%)	13 (100)
Age, years	65 ± 6.2
BMI, kg/m^2^	29.6 ± 5.6
BSA, m^2^	1.94 ± 0.2
Previous MI	13 (100)
Hypertension	12 (92)
Diabetes	5 (38.5)
Hypercholesterolemia	11 (84.6)
Peripheral artery disease	5 (38.5)
Chronic kidney disease	1 (7.7)
Previous PCI	5 (38.5)
Previous CABG	3 (23)
LAD stenosis	
CTO, *n* (%)	8 (61.5)
75-99%, *n* (%)	5 (38.5)
CCS class	
I	0
II	5 (38.5)
III	7 (53.8)
IV	1 (7.7)

All data presented as mean ± SD unless specified otherwise. BMI: body mass index; BSA: body surface area; post MI: history of myocardial infarct; HA: hypertonia arterial; DM: diabetes mellitus; post PCI: history of percutaneous coronary intervention; post CABG: history of coronary artery bypass grafting; LAD: left anterior descending artery; CTO: chronic total occlusion; CCS: The Canadian Cardiovascular Society scale.

**Table 2:** Left ventricle functional assessment by cardiac magnetic resonance.

	Baseline (*n* = 13)	12 months follow-up (*n* = 13)	*p*
LVEF [%]	36.7 ± 13.2	39.7 ± 14.9	0.052
LVEDV [mL]^∗^	230.2 (179.4-379.9)	231.7 (181.9-408.5)	0.075
LVESV [mL]^∗^	149.5 (86.2-298.5)	119.7 (81.2-314.3)	0.650
Stroke volume [mL]	83.1 ± 8.5	93.8 ± 13.8	0.025
Cardiac output [L/min]	5.0 ± 0.7	5.5 ± 0.9	0.073
Myocardial mass [mg]	192.5 ± 57.5	192.23 ± 53.7	0.974
LVEDV index [mL/m^2^]^∗^	117.7 (95.5-185.3)	120.0 (97.2-204.1)	0.152
LVESV index [mL/m^2^]^∗^	68.9 (50.2-147.2)	61.6 (45.4-161.5)	0.753
Stroke volume index [mL/m^2^]	43.3 ± 7.6	48.7 ± 9.1	0.019
Cardiac index [L/min/m^2^]	2.60 ± 0.5	2.90 ± 0.7	0.075
Myocardial mass index [mg/m^2^]	98.3 ± 26.3	98.1 ± 23.1	0.956
Body surface area [m^2^]	1.94 ± 0.22	1.95 ± 0.22	0.782
Peak ejection rate index [mL/s/m^2^]	211.0 ± 32.6	220.0 ± 28.1	0.483
Peak filling rate index [mL/s/m^2^]	176.8 ± 47.8	185.5 ± 59.2	0.573
PER/LVEDV	1.76 ± 0.6	1.78 ± 0.8	0.673
PFR/LVEDV	1.50 ± 0.6	1.45 ± 0.6	0.790

All data presented as mean ± SD unless specified otherwise. ^∗^Data presented as median with percentile: 25 and 75 LVEF: left ventricle ejection fraction; LVEDV: left ventricle end-diastolic volume; LVESV: left ventricle end-systolic volume; PER: peak ejection rate; PFR: peak filling rate.

**Table 3:** Regional myocardial thickening assessed by cardiac magnetic resonance.

Segments	Baseline		12 months follow-up		*p*
	Absolute (mm)	Relative (%)	Absolute (mm)	Relative (%)	
Basal segment					
1	2.11	20.79	1.83	20.35	0.5
2	1.15	10.59	1.18	11.26	0.75
3	1.59	18.04	1.26	12.16	0.13
4	2.69	28.99	2.66	24.84	0.13
5	4.16	46,47	3.47	35.46	0.04
6	3.48	35.88	3.47	41.71	0.75
Mid segment					
7	2.78	30.00	2.78	35.07	0.28
8	1.58	16.08	1.89	17.83	0.19
9	3.17	28.65	2.04	23.13	0.8
10	2.66	28.95	3.43	28.04	0.04
11	3.12	42.30	4.03	34.47	0.04
12	4.1	42.10	4.12	54.30	0.11
Apical segment					
13	2.13	34.71	3.02	47.68	0.34
14	0.54	9.11	2.13	28.80	0.55
15	1.22	17.49	2.08	25.00	0.05
16	0.78	11.01	3.40	54.46	0.01

## Data Availability

Source data will be deposited as a supplementary material document presenting anonymized data.

## Supplementary Materials

Supplementary Materials The “RAW DATA LASERS ADSC” was submitted as a supplementary file with raw, anonymized data that were used for calculations.

## References

[1] Ambrosy A. P., Fonarow G. C., Butler J. (2014). The global health and economic burden of hospitalizations for heart failure: lessons learned from hospitalized heart failure registries. *Journal of the American College of Cardiology*.

[2] Pikala M., Maniecka-Bryła I. (2017). Fifteen-year mortality trends due to cardiovascular diseases in Poland using standard expected years of life lost, 2000–2014. *Kardiologia Polska*.

[3] Henry T. D., Satran D., Jolicoeur E. M. (2014). Treatment of refractory angina in patients not suitable for revascularization. *Nature Reviews. Cardiology*.

[4] Jolicoeur E. M., Ohman E. M., Temple R. (2008). Clinical and research issues regarding chronic advanced coronary artery disease part II: trial design, outcomes, and regulatory issues. *American Heart Journal*.

[5] Jolicoeur E. M., Cartier R., Henry T. D. (2012). Patients with coronary artery disease unsuitable for revascularization: definition, general principles, and a classification. *The Canadian Journal of Cardiology*.

[6] Ponikowski P., Voors A. A., Anker S. D. (2016). 2016 ESC guidelines for the diagnosis and treatment of acute and chronic heart failure: the task force for the diagnosis and treatment of acute and chronic heart failure of the European Society of Cardiology (ESC) developed with the special contribution of the Heart Failure Association (HFA) of the ESC. *European Heart Journal*.

[7] Redfield M. M., Jacobsen S. J., Burnett J. C., Mahoney D. W., Bailey K. R., Rodeheffer R. J. (2003). Burden of systolic and diastolic ventricular dysfunction in the community: appreciating the scope of the heart failure epidemic. *JAMA*.

[8] Bleumink G., Knetsch A., Sturkenboom M. (2004). Quantifying the heart failure epidemic: prevalence, incidence rate, lifetime risk and prognosis of heart failure the Rotterdam study. *European Heart Journal*.

[9] Ceia F., Fonseca C., Mota T. (2002). Prevalence of chronic heart failure in southwestern Europe: the EPICA study. *European Journal of Heart Failure*.

[10] Henry T. D., Satran D., Hodges J. S. (2013). Long-term survival in patients with refractory angina. *European Heart Journal*.

[11] Maggioni A. P., Dahlström U., Filippatos G. (2013). EURO*bservational* Research Programme: regional differences and 1-year follow-up results of the Heart Failure Pilot Survey (ESC-HF Pilot). *European journal of heart failure*.

[12] Bondarenko O., Beek A. M., McCann G. P., van Rossum A. C. (2012). Revascularization in patients with chronic ischaemic myocardial dysfunction: insights from cardiovascular magnetic resonance imaging. *European Heart Journal Cardiovascular Imaging*.

[13] Knuuti J., Wijns W., Saraste A. (2020). 2019 ESC guidelines for the diagnosis and management of chronic coronary syndromes. *European Heart Journal*.

[14] Szöke K., Brinchmann J. E. (2012). Concise review: therapeutic potential of adipose tissue-derived angiogenic cells. *Stem Cells Translational Medicine*.

[15] Wang L., Deng J., Tian W. (2009). Adipose-derived stem cells are an effective cell candidate for treatment of heart failure: an MR imaging study of rat hearts. *American Journal of Physiology. Heart and Circulatory Physiology*.

[16] Yu H., Lu K., Zhu J., Wang J. (2017). Stem cell therapy for ischemic heart diseases. *British Medical Bulletin*.

[17] Konstanty-Kalandyk J., Bartuś K., Piątek J. (2018). Midterm outcomes of transmyocardial laser revascularization with intramyocardial injection of adipose derived stromal cells for severe refractory angina. *Postepy Kardiol Interwencyjnej*.

[18] Konstanty-Kalandyk J., Piątek J., Chrapusta A. (2018). Use of adipose-derived stromal cells in the treatment of chronic ischaemic heart disease: safety and feasibility study. *Kardiologia Polska*.

[19] Fraser J. K., Hicok K. C., Shanahan R., Zhu M., Miller S., Arm D. M. (2014). The Celution®System: automated processing of adipose-derived regenerative cells in a functionally closed system. *Advances in wound care*.

[20] Tylka J., Piotrowicz R. (2009). Quality of life questionnaire SF-36 -- polish version. *Kardiologia Polska*.

[21] Schenke-Layland K., Strem B. M., Jordan M. C. (2009). Adipose tissue-derived cells improve cardiac function following myocardial infarction. *The Journal of Surgical Research*.

[22] Perin E. C., Sanz-Ruiz R., Sanchez P. L. (2014). Adipose derived regenerative cells in patients with ischemic cardiomyopathy: the PRECISE trial. *American Heart Journal*.

[23] Solomon S. D., Anavekar N., Skali H. (2005). Influence of ejection fraction on cardiovascular outcomes in a broad spectrum of heart failure patients. *Circulation*.

[24] Kastrup J., Schou M., Gustafsson I. (2017). Rationale and design of the first double-blind, placebo-controlled trial with allogeneic adipose tissue-derived stromal cell therapy in patients with ischemic heart failure: a phase II Danish multicentre study. *Stem cells international*.

[25] Dobrowolska M., Miękus P., Świątczak M., Raczak G., Daniłowicz-Szymanowicz L. (2021). Two--year prognosis of patients hospitalized for decompensated heart failure in a district general hospital. *Kardiologia Polska*.

[26] Henry T. D., Pepine C. J., Lambert C. R. (2017). The Athena trials: Autologous adipose-derived regenerative cells for refractory chronic myocardial ischemia with left ventricular dysfunction. *Catheterization and Cardiovascular Interventions*.

[27] Paitazoglou C., Bergmann M. W., Vrtovec B. (2019). Rationale and design of the European multicentre study on Stem Cell therapy in IschEmic Non-treatable Cardiac disease (SCIENCE). *European Journal of Heart Failure*.

[28] Mathiasen A. B., Qayyum A. A., Jørgensen E. (2020). Bone marrow-derived mesenchymal stromal cell treatment in patients with ischaemic heart failure: final 4-year follow-up of the MSC-HF trial. *European Journal of Heart Failure*.

[29] Collinson D. J., Donnelly R. (2004). Therapeutic angiogenesis in peripheral arterial disease: can biotechnology produce an effective collateral circulation?. *European Journal of Vascular and Endovascular Surgery*.

[30] De Ugarte D. A., Morizono K., Elbarbary A. (2003). Comparison of multi-lineage cells from human adipose tissue and bone marrow. *Cells, Tissues, Organs*.

