# Supplementary material for: Functional Recovery after Intramyocardial Injection of Adipose-Derived Stromal Cells Assessed by Cardiac Magnetic Resonance Imaging
Source: Stem Cells Int. 2021 Apr 22;2021:5556800. doi: 10.1155/2021/5556800 (PMC8087467; doi:10.1155/2021/5556800)
Supplement: Supplementary Materials — The “RAW DATA LASERS ADSC” was submitted as a supplementary file with raw, anonymized data that were used for calculations. [file 5556800.f1.pdf]

ID	1	2	3	4	5	7	8	9	10	11	12	13	14
PREOP_DATA													
AGE	65,00	73,00	64,00	70,00	55,00	67,00	69,00	54,00	70,00	71,00	65,00	65,00	56,00
LVEF	32,70	21,35	17,86	18,40	23,30	52,26	48,35	52,95	35,49	42,62	35,88	43,84	52,35
EDvolume	230,16	361,50	411,98	398,37	407,85	177,14	164,99	185,42	231,67	181,60	253,83	185,16	158,48
ESvolume	154,87	284,23	338,39	325,00	312,81	84,56	85,22	87,24	149,46	104,20	162,75	103,98	75,53
Strokevolume	75,28	77,17	73,60	73,30	95,04	92,58	79,78	98,18	82,21	77,40	91,08	81,18	82,96
Cardiacoutput	3,99	4,94	4,49	3,74	6,18	5,55	5,03	5,40	5,10	4,18	5,65	5,68	4,89
Myocmassavg	266,81	246,18	261,96	172,64	227,17	127,53	187,00	83,26	181,57	266,07	170,68	153,25	158,86
EDvolumemlm2	102,45	213,22	187,63	224,59	183,01	96,38	75,17	117,71	119,27	94,63	124,93	102,57	86,92
ESvolumemlm2	68,94	167,69	154,11	183,26	140,36	46,00	38,82	55,38	76,95	54,30	80,10	57,60	41,42
Strokevolumemlm2	33,51	45,53	33,52	41,32	42,65	50,37	36,34	62,33	41,32	40,34	44,83	44,97	45,50
Cardiacindexlminm2	1,78	2,91	2,04	2,11	2,77	3,00	2,29	3,43	2,62	2,18	2,78	3,15	2,68
Myocmassgm2	118,77	145,24	119,31	97,33	101,93	69,39	85,21	52,86	93,48	138,65	84,01	84,89	87,13
FU_DATA_A													
LVEF_A	35,85	25,18	20,65	21,09	17,83	59,47	48,33	57,63	46,25	53,87	36,70	39,02	54,21
EDvolume_A	300,68	392,82	438,46	424,19	471,83	184,14	231,73	185,89	169,51	181,51	270,35	182,30	136,06
ESvolume_A	192,90	293,90	347,90	334,73	387,68	74,63	119,73	78,76	91,11	83,73	171,13	111,18	62,31
Strokevolume_A	107,79	98,91	90,56	89,46	84,15	109,52	112,00	107,13	78,40	97,78	99,22	71,13	73,76
Cardiacoutput_A	4,53	5,44	4,62	4,47	5,72	6,68	6,00	6,53	3,76	6,45	6,15	4,98	6,20
Myocmass	277,51	235,52	233,68	231,30	250,90	125,97	194,08	101,22	210,26	192,71	142,79	151,85	151,28
EDvolumemlm2_A	132,85	224,99	194,90	233,94	213,34	97,65	107,90	120,01	86,02	96,72	124,26	100,98	76,83
ESvolumemlm2_A	85,23	168,34	154,64	184,60	175,29	39,57	55,75	50,84	46,23	44,62	84,98	61,58	35,18
Strokevolumemlm2_A	47,62	56,66	40,26	49,34	38,00	58,00	52,15	69,16	39,79	52,11	49,27	39,40	41,65
Cardiacindexlminm2_A	2,00	3,12	2,05	2,47	2,59	3,54	2,82	4,22	1,91	3,44	3,05	2,76	3,50
Myocmassgm2_A	122,62	134,90	103,96	127,56	113,45	66,80	90,37	65,35	106,70	102,69	70,91	84,11	85,42
DELTAEF	3,15	3,83	2,79	2,69	-5,47	7,21	-0,02	4,68	10,76	11,25	0,82	-4,82	1,86
DELTAEDVOL	70,52	31,32	26,48	25,82	63,98	7,00	66,74	0,47	-62,16	-0,09	16,52	-2,86	-22,42
DELTAESVOL	38,03	9,67	9,51	9,73	74,87	-9,93	34,51	-8,48	-58,35	-20,47	8,38	7,20	-13,22
DELTASV	32,51	21,74	16,96	16,16	-10,89	16,94	32,22	8,95	-3,81	20,38	8,14	-10,05	-9,20
DELTAMM	10,70	-10,66	-28,28	58,66	23,73	-1,56	7,08	17,96	28,69	-73,36	-27,89	-1,40	-7,58
DELTASVI	14,11	11,13	6,74	8,02	-4,65	7,63	15,81	6,83	-1,53	11,77	4,44	-5,57	-3,85
